# *Mycobacterium tuberculosis* Specific Protein Rv1509 Evokes Efficient Innate and Adaptive Immune Response Indicative of Protective Th1 Immune Signature

**DOI:** 10.3389/fimmu.2021.706081

**Published:** 2021-07-27

**Authors:** Manjunath P, Javeed Ahmad, Jasmine Samal, Javaid Ahmad Sheikh, Simran Kaur Arora, Mohd Khubaib, Heena Aggarwal, Indu Kumari, Kalpana Luthra, Syed Asad Rahman, Seyed E. Hasnain, Nasreen Z. Ehtesham

**Affiliations:** ^1^Inflammation Biology and Cell Signaling Laboratory, National Institute of Pathology, New Delhi, India; ^2^Department of Biotechnology, Jamia Hamdard, New Delhi, India; ^3^Department of Biophysics, All India Institute of Medical Sciences, New Delhi, India; ^4^Department of Biochemistry, All India Institute of Medical Sciences, New Delhi, India; ^5^BioInceptionPvt. Ltd., Chelmsford, United Kingdom; ^6^Department of Biochemical Engineering and Biotechnology, Indian Institute of Technology, New Delhi, India; ^7^Department of Life Science, School of Basic Sciences and Research, Sharda University, Greater Noida, India

**Keywords:** B cells, dendritic cells, effector memory, human TB patients, pathogenesis, T cells

## Abstract

Dissecting the function(s) of proteins present exclusively in *Mycobacterium tuberculosis* (*M.tb*) will provide important clues regarding the role of these proteins in mycobacterial pathogenesis. Using extensive computational approaches, we shortlisted ORFs/proteins unique to *M.tb* among 13 different species of mycobacteria and identified a hypothetical protein Rv1509 as a ‘signature protein’ of *M.tb*. This unique protein was found to be present only in *M.tb* and absent in all other mycobacterial species, including BCG. *In silico* analysis identified numerous putative T cell and B cell epitopes in Rv1509. Initial *in vitro* experiments using innate immune cells demonstrated Rv1509 to be immunogenic with potential to modulate innate immune responses. Macrophages treated with Rv1509 exhibited higher activation status along with substantial release of pro-inflammatory cytokines. Besides, Rv1509 protein boosts dendritic cell maturation by increasing the expression of activation markers such as CD80, HLA-DR and decreasing DC-SIGN expression and this interaction was mediated by innate immune receptor TLR2. Further, *in vivo* experiments in mice demonstrated that Rv1509 protein promotes the expansion of multifunctional CD4+ and CD8+T cells and induces effector memory response along with evoking a canonical Th1 type of immune response. Rv1509 also induces substantial B cell response as revealed by increased IgG reactivity in sera of immunized animals. This allowed us to demonstrate the diagnostic efficacy of this protein in sera of human TB patients compared to the healthy controls. Taken together, our results reveal that Rv1509 signature protein has immunomodulatory functions evoking immunological memory response with possible implications in serodiagnosis and TB vaccine development.

## Introduction

*Mycobacterium tuberculosis* (*M.tb*), the tuberculosis (TB) pathogen, is one of the oldest pathogens in human history which infects millions worldwide. Of the over 10 million people who fall sick each year due to tuberculosis (TB), 1.2 million died in 2019 (1). Over the last five years, an increasing percentage (21%, 0.46 million) of drug resistant TB further complicates this problem (2) necessitating programmatic versus personalized approaches to manage multidrug resistant tuberculosis (MDR-TB) (3). *M.tb* has evolved multiple strategies to evade and exploit the host immune surveillance system for its survival inside the macrophages. Despite the century old Bacillus Calmette Guerin (BCG) vaccine against TB, we have still not been successful in containing the spread of TB (4). Several efforts in improving diagnostics, vaccines and treatment for TB have been impeded by the complex biology and heterogeneous interplay between the host and *M.tb.* BCG, a live attenuated strain of *M.bovis*, confers insufficient protection against pulmonary tuberculosis in adults (5) highlighting the need for identification and characterization of novel mycobacterial antigens that can induce a protective immune response against *M.tb*. Comparative computational approaches of different species of mycobacterium revealed reductive evolution along with concomitant gain in pathogenesis (6, 7). Genes playing a key role(s) in virulence and pathogenesis were retained by *M.tb*, and elucidation of functional aspects of these genes is, therefore important to understand the pathogenesis of TB. Identification and characterization of new *M.tb* antigens (termed as “hypothetical” genes with uncharacterized function in Mycobrowser (https://mycobrowser.epfl.ch/)) have been accelerated during the last few years, particularly following the computational annotation approaches. We recently showed the immunomodulatory role of a *M.tb* specific hypothetical protein, Rv1507A under *in vitro* and *in vivo* conditions, suggesting its potential in vaccine development against *M.tb* (8). Other representative mycobacterial proteins with diverse functions also modulated immune responses to potentiate the virulence of the pathogen (8–11). Several previous reports investigating relevant antigens of *M.tb* identified genes within the region of difference (RD) as potential antigens that could be promising for immune diagnosis, vaccine development and for a better understanding of the host-pathogen pathogenesis (12–15).

*M.tb* successfully invades and hijacks host machinery to dampen the host immune attack and replicates inside host macrophages. The interplay between *M.tb* and the host defense mechanisms determines the manifestations of infection. Traditionally, adaptive immunity comprising of T and B cells is responsible for protection and long-term memory response against *M.tb*, but increasing lines of evidence have underscored the significance of innate immunity in the induction of protection and memory response (16, 17). Multifunctional CD4*+*/CD8+Tcells are known to mediate protective immunity in various disease models, including TB, and induce vaccine-mediated protection (18, 19). However, *M.tb* being a successful human pathogen can delay the initiation of antigen-specific T cell response *via* impairment of dendritic cells (DC)maturation. DCs and macrophages act as immunological sensors to alert the immune system. Further, immature DCs are inefficient stimulators of T cells and need maturation that involves secretion of immunoregulatory cytokines, upregulation of co-stimulatory/adhesion molecules and loss of endocytic/phagocytic receptors to initiate the rapid and robust innate immune response. By impeding DC maturation, *M.tb* dampens CD4+ T cell responses, shaping immune response to its benefit. Therefore, mycobacterial antigens with the desired property of activating polyfunctional CD4+/CD8+T cells and serving as targets for immune system need to be identified and evaluated as possible candidate (s) to enhance the efficacy of BCG in terms of inducing long term memory response (20). In order to look for such immunogenic mycobacterial antigens, we performed a sequence comparison of all the proteins across 13 species of mycobacteria using protein BLAST tool to identify those proteins which are unique to *M.tb*. Using Nucleotide and Protein BLAST tools, we analyzed and identified one such hypothetical protein, Rv1509, as exclusive (‘signature’) to *M.tb* and absent in all other mycobacterial species. In this study, we describe the immunological attributes of this signature protein that unravel its candidature as a possible stand-alone subunit candidate vaccine or for creating a modified recombinant BCG.

## Materials and Methods

### Materials

Recombinant human IL-4, GM-CSF, fluorescent antibodies (**Supplementary Table 1**) used for DC maturation assay and anti-human/anti-mouse IgG were purchased from Biolegend (San Diego, USA). All antibodies for mice experiments were obtained from BD (BD Biosciences, San Jose CA, USA). ELISA kits for cytokine profiling were purchased from Peprotech (Rehovot, Israel).

### Comparative Proteomic Analysis of 13 Mycobacterium Species

BLASTp (http://blast.ncbi.nlm.nih.gov/Blast.cgi) of all 13 mycobacterium species proteome versus all 13 mycobacterium species (**Supplementary Table 1**) was carried out using sequence identity cut-off of 20% and e-value cut-off of 0.0001. The BLASTp results were used to create a list of protein sequences unique to *M.tb*. Nucleotide sequences of shortlisted 25 unique proteins were compared using nucleotide blast (BLASTn. http://blast.ncbi.nlm.nih.gov/Blast.cgi?PROGRAM=blastn&PAGE) with all in National Centre for Biotechnology Information (NCBI) (http://www.ncbi.nlm.nih.gov/).

### *In Silico* Structural and Functional Analysis

Different computational tools were used to understand the possible function(s) of shortlisted unique hypothetical proteins. The tools used for analysis include Expasy-Protparam (http://web.expasy.org/cgi-bin/protparam/protparam) for protein sequence analysis, Expasy-Prosite (http://web.expasy.org/cgi-bin/protparam/protparam) and Conserved Domain Database (http://www.ncbi.nlm.nih.gov/cdd) for domain and motif search. Globplot (http://globplot.embl.de/) was used for structural analysis. For secondary structure prediction Jpred (http://www.compbio.dundee.ac.uk/jpred/), MINNOU (http://minnou.cchmc.org/) for membrane protein identification and IEDB (http://tools.immuneepitope.org/bcell/) for B-cell epitope prediction. Antigenpro (http://scratch.proteomics.ics.uci.edu/explanation.html#ANTIGENpro) was used for protein antigenicity.

### T Cell and B Cell Epitope Prediction

The complete amino acid sequence of Rv1509 protein was retrieved from the NCBI database. Epitopes for T cell MHC class I and II were identified by the immune epitope database analysis resource (IEDB-AR) (21). MHC molecules should bind to processed antigenic peptide for recognition by T cells. Eight human MHC class I alleles HLA-A*0201, HLA-A*0206, HLA-A*6801, HLA-B*1501, HLA-B*3501, HLA-B*4001, HLA-B*5301 and HLA-B*5801 were selected for peptides to MHC class I epitope. The binding affinity of peptides to MHC class I molecules was predicted in terms of inhibitory concentration (IC50). Based on IC50 values, T-cell epitopes which showed IC50 value <50 classified as high-affinity molecules. Artificial neural network (ANN) method was employed to obtain nine-mer MHC class I epitopes (22–24). For MHC class II, the peptide length was 15-mer, and consensus method was used for epitope prediction with human MHC Class II alleles HLA-DR alleles DRB1*0101, DRB1*0301, DRB1*0401, DRB1*0701, DRB1*0801, DRB1*1101, and DRB1*1501 (25, 26). The consensus method employs stabilization matrix alignment method and the average relative binding matrix strategy to predict the binding of the epitopes. The obtained T cell MHC Class epitopes were subjected to BLASTp to closely related mycobacterial species to exclude cross-reactivity. Linear and conformational B cell epitopes were predicted using the ElliPro suite (27), which implemented Thornton’s method MODELLER (28) and the Jmol viewer (29) program were used to obtain 3D B-cell epitopes. Threshold values for epitope analysis were the score set 0.8 and 6Å maximum distance for residues clustering and selected to obtain the epitopes. Furthermore, the obtained T cell MHC class and B cell peptide epitopes were subjected to BLASTp analysis at NCBI against the proteome of closely related mycobacterial species for identifying any overlapping epitopes to be excluded in order to avoid any existing cross-reactivity.

### Molecular Docking of the Epitopes Binding to HLA Alleles

HLA DRB allele crystal structures of human were retrieved from the PDB. The endogenous antigenic peptides were excluded from the crystal structure of HLA-A and HLA-DRB alleles and defined as a receptor. The B cell and T cell epitopes were docked manually into the homologous ligand position from the HLA crystal structures with PDB id 1JF1, 3OXR, 1AQD and 1XR8. The docking was carried out using coordinates replacement into the protein using Pymol and Coot 0.7.2 (30, 31). Peptide epitopes were obtained from the modelled protein structure of Rv1509 and aligned with the peptide of the crystal structure to obtain aligned ligand. The HLA protein structure and aligned ligand were merged using Coot-.7.2 (31). The native protein structure and protein-peptide structure was energy minimized using GROMACS 5.0 (GROningen MAchine for Chemical Simulations) package using the OPLS force field (32) using steepest descent and conjugate gradient method. The protein-peptide interactions were analyzed by LigPlot+ (33).

### Structural Modeling Of Rv1509 and Its Interactions With TLR2 Complex

The amino acid sequence of *M.tb* Rv1509 was retrieved from the UniProtKB database. The retrieved amino acid sequence was subjected to homology modelling using Phyre2 server and 3D structure of the protein was obtained (34). The final model was also checked for its quality using Ramachandran plot by Coot 0.7.2 (31). The molecular interactions were analyzed by ClusPro server and the model obtained after docking was checked for protein-protein interactions using LigPlot^+^ (33).

### Cloning, Expression, and Purification of Signature Protein Rv1509

Rv1509 hypothetical gene was PCR-amplified from *M.tb* DNA using gene-specific forward and reverse primers 5’TTAAGCTTGTAATGGTGTTTGCGTTGAG3’ and 5’ATCTCGAGTTACCTCTTCGTTAGCCGCAC3’, respectively. PCR amplified product was ligated into the expression vector pET28a using *Bam*HI and *Hin*dIII restriction sites. The recombinant protein was expressed in Endotoxin free Clear Coli (BL21-DE3)and purified by Ni-NTA chromatography using N-lauryl-sarcosine denaturant as a solubilizing agent. The purified protein was dialyzed in buffer containing, 1X PBS and 10% glycerol. Protein was concentrated using 3kDa cut off centricons. Purified protein was analyzed using SDS-PAGE followed by western blotting. Protein estimation was carried out using BCA protein estimation kit as per the manufacturer’s instructions (Thermo Scientific, Massachusetts, USA).

### Cytokine Estimation From RAW264.7 and TLR Mutant Macrophage Cells

Estimation of cytokines and identification of TLR interaction study was designed and performed based on previously used method from our lab (35). These TLR knockout cell lines were obtained from BEI Resources established by the National Institute of Allergy and Infectious Diseases (NIAID) Maryland, USA. RAW264.7, ΔTLR4 and ΔTLR2/4 mouse macrophages were seeded (0.3×10^6^ cells per well) in 24 well tissue culture plates and incubated for 4 hours at 37° C for adherence. Then cells were treated with different concentrations of protein (1μg/ml, 2μg/ml and 4μg/ml) or lipopolysaccharide (LPS) (50ng/ml; Positive control) (Sigma USA) along with Control (no protein). After 48 hours of treatment, supernatants were collected and cytokines were estimated in culture supernatants. The estimation of pro-inflammatory cytokines such as TNFα, IL-12 and IL-6, and anti-inflammatory cytokine IL-10 was performed using mouse ELISA kit following the manufacturer’s instructions.

### Generation of Polyclonal Antibodies to Rv1509

Polyclonal antibodies against purified Rv1509 protein were generated in-house in rabbits by subcutaneous injection of 200μg/ml of purified protein emulsified with an equal volume of Freund’s incomplete adjuvant (Sigma-Aldrich, St. Louis, MO, USA). Two booster immunizations were administered after first dose at 15-day intervals. The antibody titer in the serum was determined by dot-blot technique two weeks post final immunization.

### Immunofluorescence Assay to Determine Interaction of Rv1509 With Cognate TLR Receptor

The macrophage cell lines ΔTLR4, ΔTLR2/4 and RAW264.7 cells were seeded (0.2 million cells/well) in 24 well plate on sterile coverslips. After 12 hours of incubation, cells were treated with Rv1509 protein at a concentration of 10μg/ml for 6 hours followed by fixing cells in 4% formaldehyde. The cells were washed three times with PBS+ 0.5% BSA. Further, cells were treated with anti Rv1509 antibody (1:100) raised in rabbit (in-house) overnight at 4°C. The cells were washed and treated with Alexa Fluor™ 594 conjugated anti-rabbit secondary antibody (1:1000). Finally, cells were mounted in Glass Antifade mountant (Thermo Fisher Scientific). Images were acquired (100X) using Olympus fluorescence microscope and processed using a freely available online software (pinetools.com).

### Immunization of Mice

Swiss albino mice (3-4 weeks) were immunized with 20 μg/ml of recombinant purified Rv1509 protein without any adjuvant to minimize the adjuvant mediated biasing of immune response towards Th1 or Th2 (36, 37) A booster dose of this protein (20 μg/ml) was administered after 15 days of primary immunization and sacrificed after 10 days of 2^nd^ booster dose.

### Human Study Population and Samples

Blood from TB uninfected healthy volunteers with clear chest X-ray and no exposure to TB patient or sample was obtained from All India Institute of Medical Science blood bank for isolation of peripheral blood mononuclear cells (PBMCs). Serum samples from healthy human donors, used as controls for B-cell response, were obtained from ICMR-National Institute of Pathology and All India Institute of Medical Sciences.

### TB Patients

Serum samples were obtained from the blood of TB patients recruited at Mahavir Hospital and Research Centre, Hyderabad, India. Different categories of TB patients were selected for analysis which included, pulmonary tuberculosis (PTB), extrapulmonary tuberculosis (EPTB), TB relapse cases and contacts. Tuberculosis in these patients was diagnosed by clinical examination, X-ray and AFB staining. Healthy controls for serum IgG quantification were uninfected. All patients and healthy donors involved in the analysis were HIV negative. Institution Bioethics Committee approved the study.

### Generation and Culture of Dendritic Cells

Dendritic cells were generated from PBMCs as described previously (38). Briefly, PBMCs were isolated from buffy coat after differential centrifugation. Monocytes were isolated by density gradient centrifugation and differentiated into immature DCs by culturing them for 7 days in RPMI-1640 containing 10% FCS, GM-CSF (1000 IU/10^6^ cells), 50U/ml penicillin G and 50 mg/ml streptomycin. Immature 7-day-old DCs (0.5×10^6^ cells/ml) were treated with PBS, LPS and recombinant Rv1509 for 48hrs. The purity of DCs was analyzed using CD11c antibody. More than 90% of DCs were found to be pure and mature as determined by FACS analysis.

### Flow Cytometric Analysis

Flow cytometric analysis was performed to analyze the expression of surface phenotypic markers like CD80, CD86, MHC-II and DC-SIGN on RAW264.7 macrophages and *in vitro* generated dendritic cells using fluorescent-tagged antibodies. For single-cell preparation of splenocytes, spleens were removed from sacrificed Swiss albino mice, crushed/perfused and passed through a syringe. Cells were washed, centrifuged and resuspended in DMEM media supplemented with RBC lysis buffer. Finally, cells were resuspended in complete DMEM media. Flow cytometric analysis was performed for CD4+/CD8+ T cell response. Splenocytes (1×10^6^/well) seeded in 96-well plate were re-stimulated with 4μg/ml protein antigen and PBS alone (control cells) for 8 hrs in the presence of monensin (BD) Golgi plug™ and Golgi stop™. T cells were collected, washed with PBS and stained with anti-CD3 (FITC), anti-CD4 (APC-H7) and anti-CD8 (PerCP/Cy5.5) fluorescent antibodies. Cells were then fixed with 4% paraformaldehyde, permeabilized in 0.02% Triton X-100, followed by washing and staining with anti-IFNγ (APC) and anti-TNF-*α* (PE-Cy7) antibodies suspended in FACS buffer (1X PBS+2% FBS+0.1% Sodium Azide +2mM EDTA) for 1 hr. Stained cells were acquired in FACS Canto II cytometer (BD Biosciences) and analyzed using FlowJo™ software.

### Analysis of Memory T Cells

Isolated splenocytes were seeded in 24 well plates and incubated with 20μg/ml of signature protein for 72hrs. Cells were washed, harvested and stained with fluorescent antibodies against memory markers (CD44 and CD62L).

### Cytokine Measurement

Levels of various cytokines in supernatants of splenocytes, induced in response to recombinant protein Rv1509 and PBS as control, were measured as described earlier (14).

### Evaluation of IgG Immune Reactivity in Immunized Mice

Immunoglobulin G reactivity against the recombinant signature protein Rv1509 using the sera collected from immunized mice was performed using ELISA (39). Briefly, Rv1509 signature protein was coated in 96 well plates overnight. The plate was washed three times with wash buffer and blocked for an hour at room temperature. After 3 washes serum samples in 1:100 dilutions were added and incubated for 2 hrs. The secondary conjugated antibody was added in 1:5000 dilution and incubated for an hour. The plate was washed at least five times, TMB substrate was added, and the reaction was stopped with 2N H_2_SO_4_. Plates were read at 450 nm with correction at 570 nm on an Eon microplate reader (Biotek, Vermont, USA) and analyzed with an in-built software (Gen5).

### Serological Characterization of Shortlisted Signature Protein Rv1509 Antigen From Tuberculosis Patient Serum

Serological analysis of Rv1509 was evaluated to check IgG immunoreactivity from sera according to the protocol described previously (40).

### Statistical Analysis

Data were analyzed using GraphPad Prism5 software. Statistical significance was determined using ANOVA and Mann-Whitney test. p< 0.05 was considered statistically significant, *p< 0.05, **p< 0.01 and ***p< 0.001.

## Results

### Comparative Analysis of Mycobacterial Genomes Reveals Proteins That Are Unique to *M.tb*

**Figure 1:**
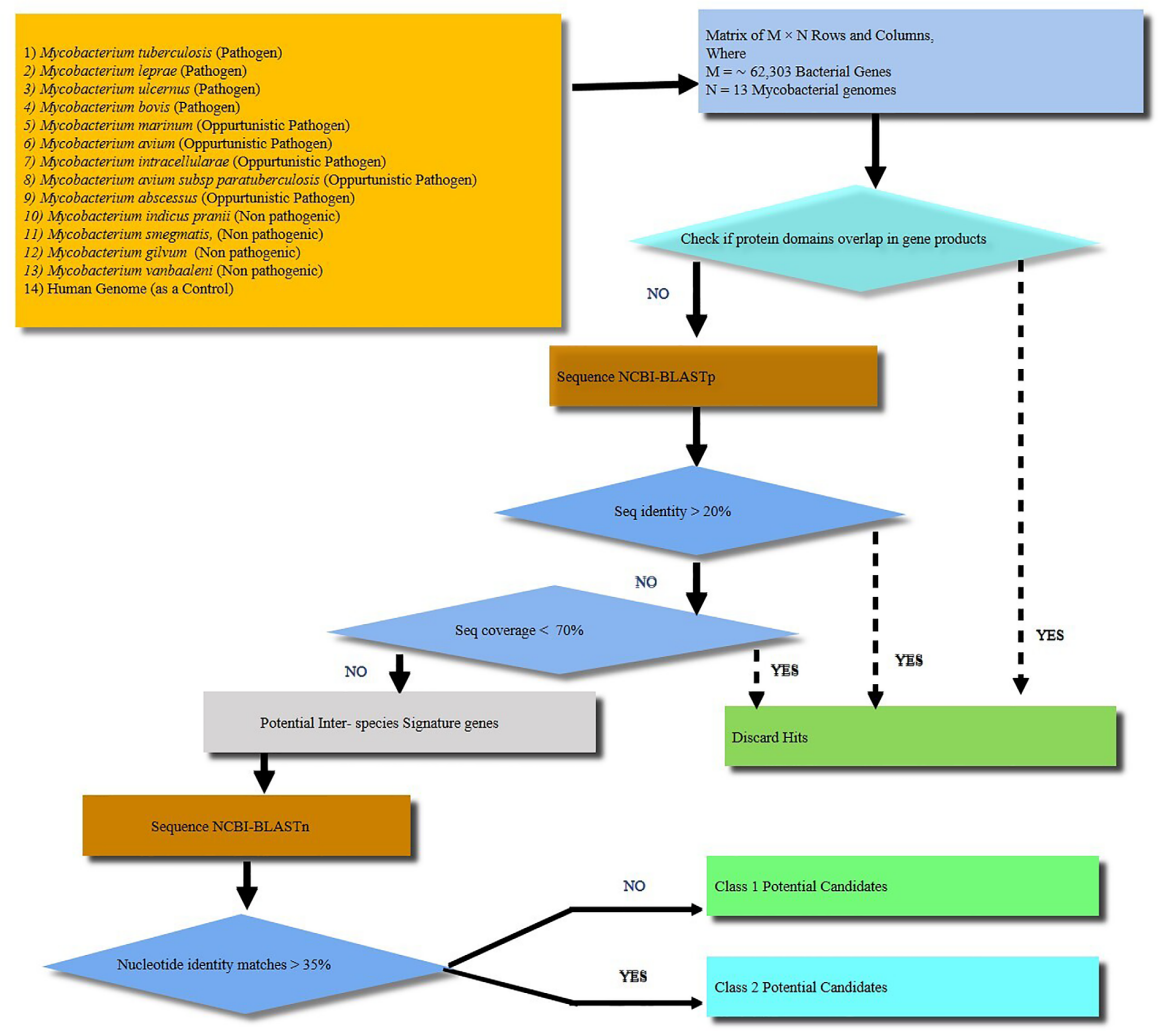
A schematic workflow showing identification of signature proteins of *Mycobacterium tuberculosis* (*M.tb*) based on computational analysis by comparing 13 mycobacterial species including strict pathogens, opportunistic pathogens and non-pathogenic mycobacteria. Human genome was taken as a control. A total of about 62,303 genes were analyzed. The *M. tb* genes with overlapping/similar protein domains to other species were omitted for further analysis. The rest of genes were then checked for protein sequence similarity analysis using protein BLAST (BLASTp). All the genes with protein sequence identity less than 20% were further analyzed for nucleotide sequence similarity. Nucleotide sequence similarity was analyzed using BLASTn and genes with less than 35% of nucleotide similarity were considered as potential signature candidates. Finally, out of the potential candidates Rv1509 emerged as a ‘signature’ of *M.tb*.

Comparative proteomic analysis of 13 mycobacterial species (**Figure 1** and **Supplementary Table 2**) revealed 25 protein sequences unique to *M.tb* which include 9 from the toxin-antitoxin category, 9 hypothetical proteins, 3 as possible prophages, 2 as acid and phagosome regulated proteins and 2 belonging to PE_PGRS family of proteins (**Supplementary Table 3**). Using nucleotide blast tool (BLASTn), we found that out of 25 selected target nucleotide sequences, 19 have their homologs in BCG. Only 6 were found to be unique to *M.tb* at both nucleotide and protein sequence level (**Supplementary Table 4**). Among the 6 unique proteins, 3 belong to prophage category, and one is found to be deleted in some clinical strains. BLAST analysis with all the available protein sequences in NCBI showed that two of these proteins, Rv1507A and Rv1509 were hypothetical and unique to *M.tb* and were therefore given the name ‘signature proteins’ of *M.tb*. The immune modulatory role of Rv1507A has been recently published (8). We identified the Grand average of hydropathy and Instability index of Unique Proteins and found that Rv1509 is a hydrophobic protein and belongs to unstable protein category (**Figure S1A, S1B**). Secondary structure prediction (**Figures S1C, D**) showed a number of alpha-helix and beta sheets. Analysis of subcellular localization of a protein is critical to understand its function. To predict the subcellular location of Rv1509, we used different web-based prediction tools (summarized in **Table 1**). To validate the predictions, we performed a western blot to determine the location of Rv1509 protein using different *M. tb* H_37_Rv extracts (total membrane, cell wall, cytosol, culture filtrate and total cell lysate). We found that signature protein Rv1509 is predominately found in the total membrane fraction of *M.tb* lysate and partially localized in the cytosol too. (**Figure S1E**).

**Table 1:** Prediction of subcellular localization by *in silico* analysis of Rv1509 protein.

S. No.	Tool used	Prediction	Score
1	TBpred	Cytoplasm	0.705
2	TBpred	Integral Membrane	0.962
3	Gpos-PLoc	Cytoplasm	
4	PSORTb	Cytoplasm	7.5

### Prediction of Putative B Cell and T Cell Epitopes, and Molecular Docking of Rv1509 Peptides With MHC Molecules

**Figure 2:**
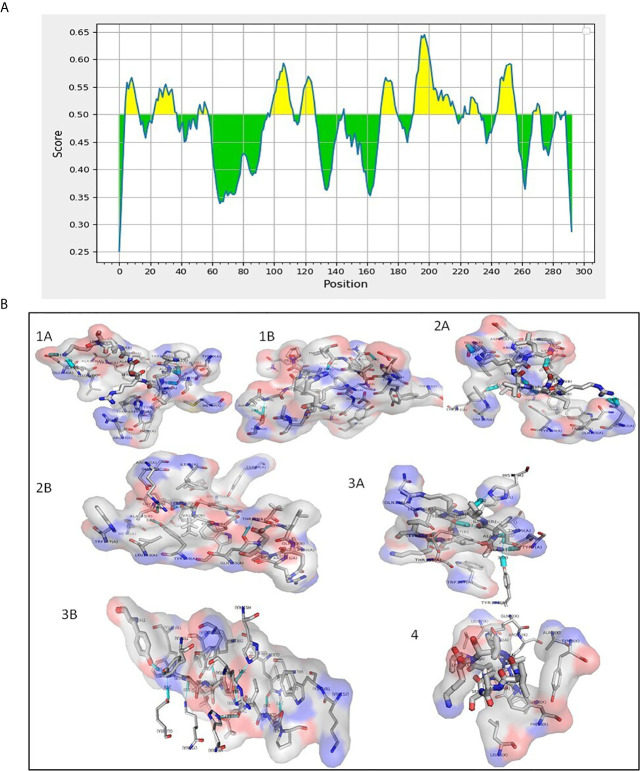
**(A)** Predicted B cell epitopes of Rv1509 protein (yellow color). B cell epitopes present in Rv1509 were predicted using IEDB web-based tools. Protein sequence corresponding to Rv1509 as obtained from Mycobrowser was analyzed by Bepipred Linear Epitope Prediction 2.0. The Y-axes depicts for each residue the correspondent score while the X-axes depicts the residue positions in the sequence. The larger score for the residues is interpreted as a higher probability to be part of epitope (yellow color on the graphs). **(B)** Interaction study of Rv1509 epitopes with MHC I and MHC II (Cyan blue-Hydrogen bonding). **(1A)** HLA*A0201 allele interactions with AAIRSAVKL (T cell MHCI & II) epitope. **(1B)** HLA*A0201 allele interactions with AGYVPATLQP (B cell and MHCI) epitope. **(2A)** HLA*B1501 allele interactions with AAIRSAVKL (T cell and MHCI & II) epitope. **(2B)** HLA*B1501 allele interactions with AGYVPATLQP (B cell and MHCI) epitope. **(3A)** HLA*A0206 allele interactions with AAIRSAVKL (T cell and MHCI & II) epitope. **(3B)** HLA*A0206 allele interactions with AAIRSAVKL (B cell and MHCI) epitope. **(4)** HLA*DR1 allele interactions with AAIRSAVKL (MHCI and MHCII) epitope.

Membrane proteins play vital role in the interactions between pathogen and the host. Hence, we were interested to understand the role of this unique protein in host-pathogen interactions. We analyzed T cell and B cell epitopes present in Rv1509 using IEDB web-based tools (**Figure 2A**). We found a total of 18 epitopes for B cell binding and 10 T cell binding epitopes (**Supplementary Tables 5–7**). The prediction showed 13 linear and 6 conformational B cell epitopes in Rv1509 (**Supplementary Tables 7, 8**). The 6 conformational B cell epitopes and their interactions with Rv1509 protein are shown (**Figure S2**). We found 6 MHC Class II binding epitopes and 4 MHC Class I binding epitopes based on the binding affinity of these molecules. It was observed that the segment of amino-acids of two epitopes YVPATLQP and VFPY were shared among B cell receptor and T cell MHC Class I epitopes (sequence highlighted). Further, AAIRSAVKL was found to be common between MHC Class I and MHC Class II epitopes (**Supplementary Tables 7, 8** and **Figure 2**). In order to improve the accuracy of prediction of epitopes, we performed molecular docking analysis of predicted epitopes with their predicted MHC molecules (**Figure 2B**). Molecular docking studies of AAIRSAVKL and AGYVPATLQP epitopes showed good binding interactions with HLA-A*0201, HLA-A*0206 and HLA-B*1501 and HLA-DRB1 of MHC Class I and II alleles of human (**Figure 2**).The predicted epitopes showed hydrogen bond and hydrophobic interactions with the HLA alleles

### Macrophages and Dendritic Cells Exhibit Enhanced Activation Upon Treatment With Rv1509 Protein

**Figure 3:**
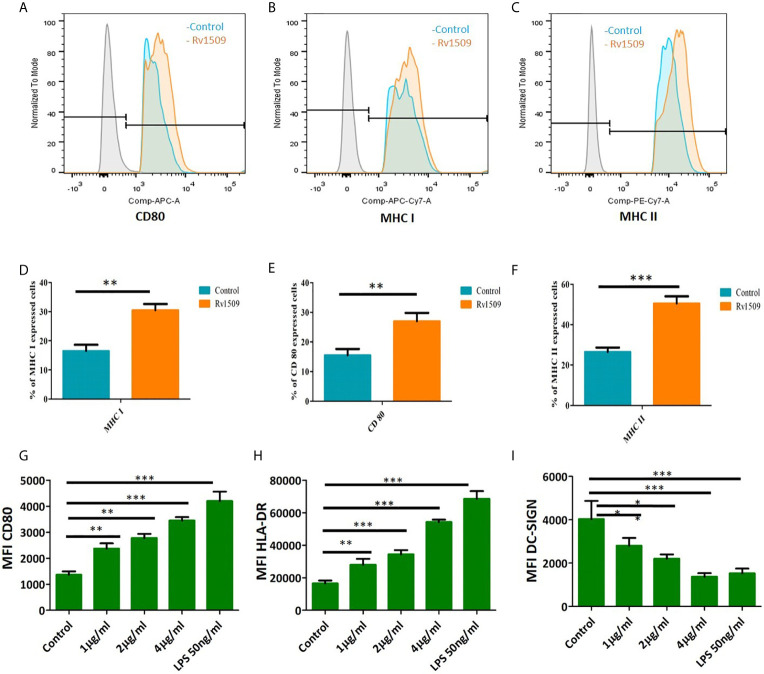
Signature protein Rv1509 induces maturation of dendritic cells and activates macrophages **(A–C)** Macrophages were treated with different concentrations of Rv1509 protein (1, 2 and 4µg/ml) followed by staining with CD80, MHC I and MHC IIafter 48 hrs of treatment. Cells were analyzed by flow cytometry and representative FACS plots depict expression of CD80, MHC I and MHC II activation markers upon treatment with Rv1509 protein in comparison to control (no protein). **(D–F)** The Graphs represent the average of percentage of CD80, MHC I and MHC II stained cells in Rv1509 treated and control macrophage cells. Seven day old immature DCs (0.5×10^6^cells) were cultured with GM-CSF and IL-4 alone (control), GM-CSF, IL4 and different concentrations (1, 2 and 4µg/ml) of Rv1509 protein, PBS and LPS (positive control) for 48 hrs and analyzed for expression of surface markers. **(G)** Maturation marker CD80 expression is increased in Rv1509 treated cells. **(H)** Expression of HLA-DR is increased in Rv1509 treated cells. **(I)** DC-SIGN expression after treatment with Rv1509 protein. Note the decreased expression of DC-SIGN in Rv1509 treated cells. Representative data are from three individual experiments with samples being treated and analyzed in triplicates at each time point. [The data are expressed as mean ± standard deviation (SD)] *p < 0.05, **p < 0.01 and ***p < 0.001.

We treated RAW macrophages with different concentrations of purified Rv1509 protein. MTT assay was performed after 24 hours of treatment to check the toxicity of this protein and we found that this protein is not toxic up to 4µg/ml (**Figure S3C**). Since our *in silico* studies indicated Rv1509 protein to be an antigenic membrane protein, we first determined whether Rv1509 protein could induce macrophage activation. Macrophage activation is very important for the innate and adaptive immune system to clear the pathogen and activate further downstream pathways. Therefore, to investigate the macrophage activation markers, we treated RAW cells with Rv1509 protein at concentrations of 1µg/ml, 2µg/ml and 4µg/ml for 48 hours. Cells were harvested and stained with macrophage surface markers such as CD80, MHC I and MHC II using specific FACS antibodies. The analyzed FACS plots reveal that Rv1509 protein significantly induces expression of activation markers on macrophages as compared to the control (**Figures 3A–C**). Calculating the percentage of CD80, MHC I and MHC II positive cells (**Figures 3D–F**) after analysis of FACS data revealed significantly increased expression in Rv1509 treated cells compared to control cells (The data are expressed as mean ± SD) (*p< 0.05, **p< 0.01 and ***p< 0.001). Nevertheless, with increasing protein concentrations, there was no significant difference in the population of activated macrophages [Data shown for the representative concentration of 2 µg/ml (**Figures 3A–F**)].

Further, human monocytes were purified from PBMCs and differentiated into DCs using GM-CSF and IL-4 (**Figure S3A, B**). The effect of Rv1509 on DC maturation was then analyzed by treating immature DC culture with different concentrations of Rv1509, or LPS treatment as a positive control, and PBS as a negative control. After 48hrs of protein treatment, the expression levels of different maturation markers such as CD80 and MHC-II were analyzed using flow cytometry. DC-SIGN, a major mycobacterial receptor on human DCs, was also analyzed for its expression in the presence and absence of Rv1509. Significant upregulation of CD80 and MHC-surface markers in cells treated with the purified Rv1509 protein was observed (**Figures 3G, H**). However, DC-SIGN expression was found to be down-regulated in Rv1509 treated cells as a direct function of protein concentration (**Figure 3I**). No such pattern could be seen in buffer treated and untreated cells (The data are expressed as mean ± SD) (*p< 0.05 and **p< 0.01). These results suggest that the signature protein enhances the levels of functional DCs by inducing maturation markers and downregulates the expression of DC-SIGN, an important receptor for the entry of *M.tb* (41).

### Rv1509 Protein Induces Production of Proinflammatory Cytokines Through TLR 2 Mediated Pathway

**Figure 4:**
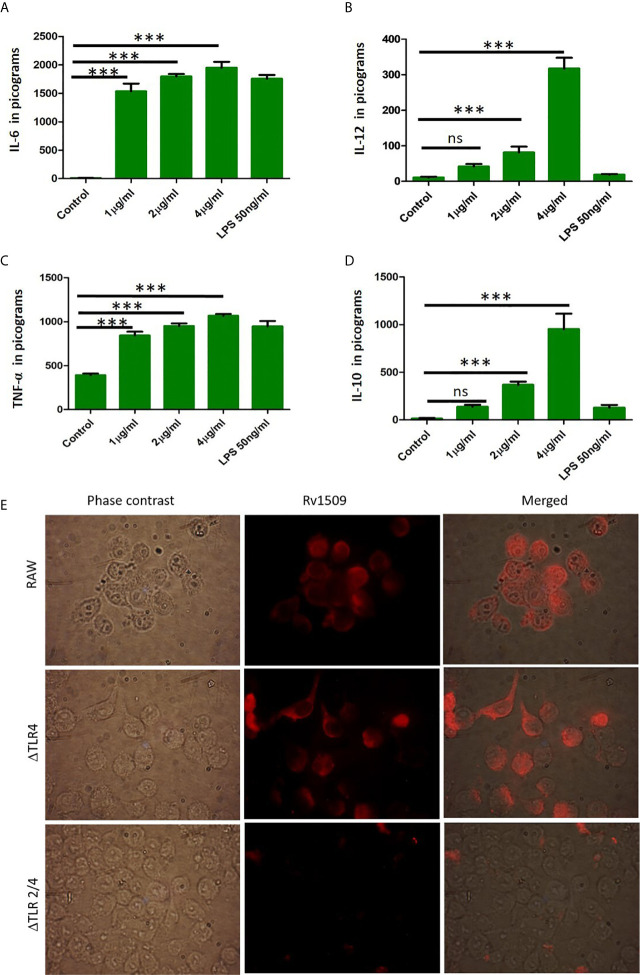
Cytokine secretion by RAW264.7 cells upon treatment with Rv1509 protein. **(A–C)** RAW 264.7 cells were seeded (0.3×10^6^cells/well) in a 24 well plate. After 12 hours of incubation cells were treated with Rv1509 protein in a dose dependent manner(1μg, 2μg and 4μg/ml) along with positive control (LPS) and negative control (no protein). The supernatants were collected 48 hrs post treatment and cytokines were estimated. The quantified data are presented as bar graphs. IL-6, IL-12 and TNFα (pro inflammatory cytokines) depicted increase in production against Rv1509 protein in a dose dependent manner compared to the control (no protein) and LPS (positive control). **(D)** The bar graph represents the secretion of anti-inflammatory cytokine IL-10 by RAW 264.7 cells upon treatment with Rv1509 protein. Representative data is from three individual experiments with minimum triplicates in each. (The data are expressed as mean ± SD). ***p < 0.001. **(E)** Immunofluorescent images of ΔTLR4, ΔTLR2/4 and RAW264.7 cells treated with Rv1509 protein (10µg/ml) and its localization in these macrophages. The cells were fixed after 6hrs of protein treatment followed by staining with anti Rv1509 antibody (prepared in-house). AlexaFluor 594 (Anti rabbit IgG) used as a detection antibody. Images were acquired using Fluorescent microscope (Olympus) at 100X (Oil immersion) magnification. ns, non-significance.

TNF-α, IL-6 and IL-12 are proinflammatory cytokines that play an important role in activation of macrophages and regulate cell death pathways. We further wanted to delineate the function(s) of Rv1509 in modulating innate immune response in terms of secretion of cytokines. We treated RAW macrophages with Rv1509 protein at concentrations of 1µg/ml, 2µg/ml and 4µg/ml and the supernatants were collected 24 hours and 48 hours post treatment. The levels of secreted cytokines were measured in the supernatants using ELISA. Results revealed that Rv1509 protein induced secretion of proinflammatory cytokines like TNFα, IL-6 and IL-12 (**Figures 4A–C**). Rv1509 also induced production of IL-10, in a concentration dependent manner, into the cell culture supernatant as compared to the untreated control cells. (The data are expressed as mean ± SD) (*p< 0.05, **p< 0.01 and ***p< 0.001, **Figure 4D**). Molecular docking of Rv1509 protein with TLR2 receptor showed that the Rv1509 potentially interacts with ligand-binding pocket of TLR2 dimer. The model which scored top from ClusPro docking of (TLR2)_2_-(Rv1509)_2_ heterotetramer was used for the analysis. It was observed that TLR2 interacted with the α-helices and loops of Rv1509 protein (**Figure S4A**). Next, we investigated whether Rv1509 mediates its function *via* TLR2 pathway as suggested by *in silico* analysis. Immunofluorescent studies to check the interactions of this protein with its respective TLR revealed that Rv1509 protein binds to TLR2 and mediates the immune modulation (**Figure 4E**).This basic experiment clearly demonstrated that Rv1509 possibly interacts with TLR2 as there was no fluorescent signal when TLR2 knockout cells were used. These results were further validated by estimating the cytokine production in ΔTLR4, ΔTLR 2/4 and RAW cells. The abrogation of cytokine production in ΔTLR2/4 demonstrates that Rv1509 protein mediates immunomodulation through TLR2 in macrophages (**Figures S4B–D**).

### Immunization of Mice With Signature Protein Rv1509 Induces Polyfunctional T Cell Subsets and Generates an Effector Memory Response

**Figure 5:**
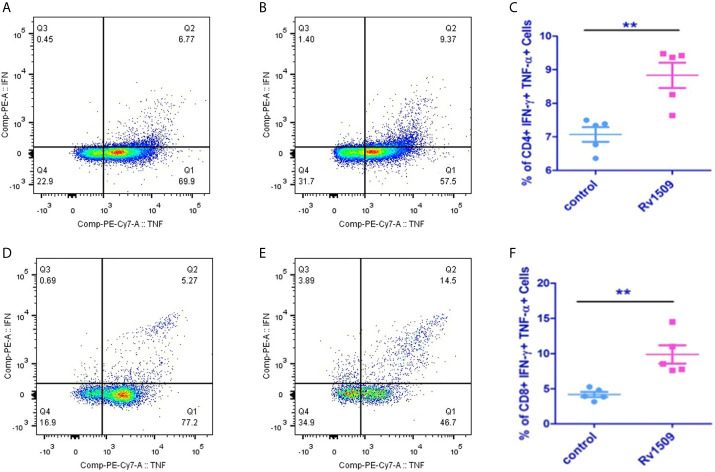
Recombinant Rv1509 protein increases number of poly-functional IFNγ and TNFα secreting CD4+ and CD8+ T cells. Splenocytes from immunized and control mice were re-stimulated with polymyxin treated recombinant Rv1509 protein in culture in the presence of Golgi plug/Golgi stop solution for 8 hours. Cells were washed, harvested and labelled for CD4+/CD8+ surface marker and intracellular IFNγ and TNFα. FACS analysis was performed by selecting splenocyte population followed by gating for CD3 initially. Selected population was then double gated to reveal CD4+ and CD8+ population. The CD4+ and CD8+ populations were then individually gated for IFNγand TNFα. The Dot plots represent the percentage population of polyfunctional T cells (IFNγ+TNFα positive cells) from a single experiment, representative of total of two experiments. **(A, B)** Representative FACS plot of control and Rv1509 protein treated cells. **(C)** Rv1509 treatment increases the number of CD4+ cells double positive for IFNγ and TNFα. **(D–F)** FACS plots and graph represent the CD8+ T cells in control and Rv1509 immunized animals after *in vitro* stimulation with protein. Rv1509 protein treatment results in an increase in number of CD8+ T cells double positive for IFNγ and TNFα. $Representative data are from five samples in each group. (The data are expressed as mean ± SD). **p < 0.01.

Based on our predictions using immunoinformatic approaches and cell line-based results, we further evaluated the nature of immune responses and characterized the phenotype of immune cells induced by immunization with recombinant Rv1509 protein. Multiparameter flow cytometry was performed with splenocytes isolated from immunized mice. The percentage of multifunctional T cells that produced IFNγ and TNFα was measured. In response to *in vitro* protein stimulation, increased numbers of double-positive (IFNγ and TNFα) CD4+T cells (**Figures 5A–C**), as well as CD8+ T cells, were observed in the cultured splenocytes isolated from the immunized mice. Much higher CD8+ polyfunctional T cells were induced as compared to CD4+ T cell subset (**Figures 5D–F**). These observations demonstrate that this protein induced an increase in the number of antigen-specific multifunctional CD4+ and CD8+T cells in mice, implicating its role in evoking protective immune response against *M.tb.*

**Figure 6:**
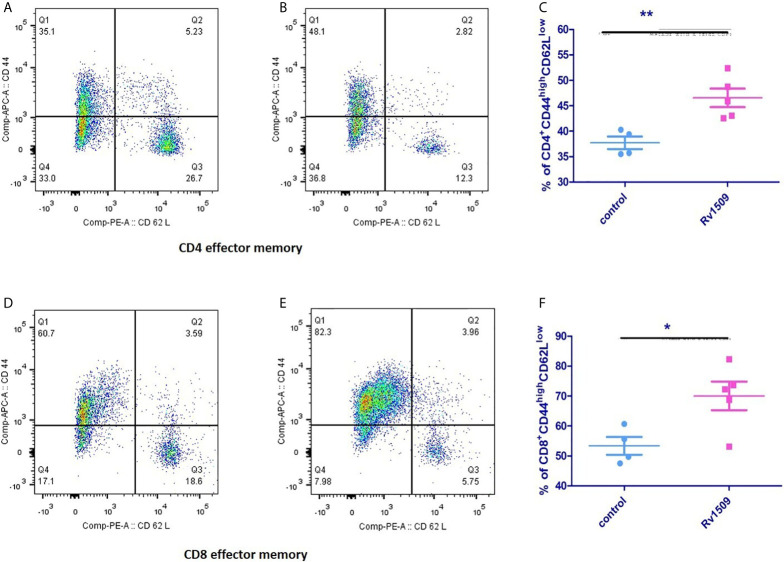
Rv1509 protein enhances effector memory cells in immunized mice. Mice (n = 5) were immunized with recombinant Rv1509 protein. Splenocytes from immunized mice were stimulated in *vitro* with rRv1509 overnight and stained with antibodies against CD3, CD4, CD8 CD44 and CD62L.Lymphocyte cells were selected and gated for CD3 population followed by CD4 and CD8 selection by double gating strategy. From CD4 and CD8 population gating was performed to identify cells expressing CD44 and CD62L. Both CD4+ and CD8+ effector memory cells (CD44^high^CD62L^low^) were increased after immunization with Rv1509 antigen. Note the enhanced number of CD4+ **(A–C)** and CD8+ **(D–F)** effector memory cells in mice immunized with Rv1509 protein antigen compared to control. Representative data are from five samples in each group from a single experiment representative of two experiments. (The data are expressed as mean ± SD).*p < 0.05 and **p < 0.01.

Next, we investigated the effect of Rv1509 on stimulating memory cell response of each T cell subset (CD4+ and CD8+) (**Figures S5A–E**). We assessed the surface phenotypic T cell memory markers, CD44 and CD62L, in immunized mice using flow cytometry. For CD4+ T cells, the effector memory T cell (T_EM_) population was significantly increased in immunized mice as compared to controls (**Figures 6A–C**). Interestingly, CD8+T cell population also showed a significantly increased T_EM_ population compared to the control cells (**Figures 6D–F**). Taken together, these findings suggest a novel role for this signature protein Rv1509 in the activation of adaptive immunity along with generation of memory response that support its possible utility as a subunit vaccine candidate (The data are expressed as mean ± SD, **p< 0.01).

### Rv1509 Protein Activates Cell Mediated as Well as Humoral Immunity

**Figure 7:**
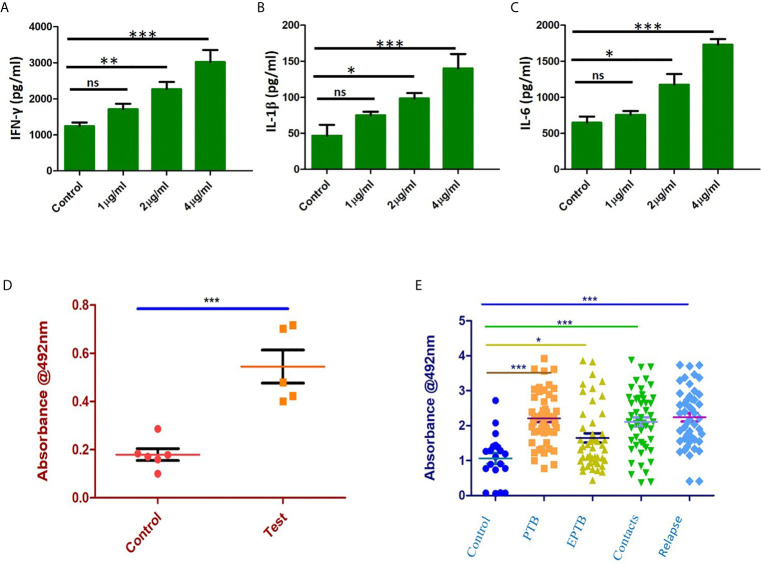
Rv1509 enhances secretion of Th1 cytokines in re-stimulated splenocytes and elicits strong B cell response in human TB patients. **(A)** Splenocytes were re-stimulated with Rv1509 in culture for 72 hrs. Supernatant from the culture was collected for sandwich ELISA. Significant increase in the level of IFNγ as a direct function of protein concentration could be seen along with IL1β **(B)** and IL-6 **(C)**. **(D)** B-cell response against Rv1509 shows a significant response in immunized mice. Serum from immunized and control animals was added to ELISA plate coated with 10 µg/ml of recombinant Rv1509. After incubation, plates were washed and bound total IgG was detected by enzyme labelled secondary anti-mouse IgG. O.D (Optical Density) is plotted as dot plots with each dot representing an animal. The data presented are from five animals in each group from a representative experiment. The increase in OD is statistically significant in case of immunized animals as compared to controls (p < 0.001). **(E)** Immunoreactivity of sera from different categories of TB patients with Rv1509 protein was studied using ELISA. Statistically significant immunoglobin IgG response was elicited by Rv1509 against control (n = 20), PTB (n = 40), EPTB (n=40), contacts (n = 40) and relapse (n = 40) cases of tuberculosis. *p < 0.05, **p < 0.01 and ***p < 0.001. ns, non-significance. (The data are expressed as mean ± SD).

Immunized mice were sacrificed, and splenocytes were isolated and cultured with different concentrations of recombinant purified Rv1509 protein. The supernatant was collected after 72hrs of incubation and assayed for different pro-inflammatory (IFNγ, IL1β and IL-6) and anti-inflammatory cytokine (IL-10) levels by ELISA. We observed a significant increase in the levels of pro-inflammatory cytokines - IFNγ (**Figure 7A**), IL1β (**Figure 7B**) and IL-6 (**Figure 7C**). These results indicate that Rv1509 induces Th1 response, a hallmark in controlling mycobacterial infection.

*In silico* analysis pointed to the presence of B cell epitopes in Rv1509 protein we therefore assessed the immunoglobulin G response (IgG) to Rv1509 protein. Serum IgG levels to *M.tb* specific antigens can have a value as serodiagnostic markers in the diagnosis of active pulmonary tuberculosis. Mice were immunized with purified Rv1509 protein, and serum IgG levels were estimated using ELISA. A significant increase in total IgG titers was seen in mice immunized with Rv1509 as compared to the controls (**Figure 7D**). This observation when viewed in the context of the fact that the signature protein is absent in other species of mycobacteria, especially BCG that is used as a vaccine, points to its potential to act as an immunodiagnostic biomarker for tuberculosis. For further validation, the IgG titers corresponding to Rv1509 protein were evaluated using human TB patient sera by ELISA. The humoral immune responses directed against the purified Rv1509 protein in pulmonary tuberculosis patients(n=40), pulmonary relapse cases(n=40), extra pulmonary tuberculosis patients (n=40) and healthy controls(n=20) were compared. Results revealed that the sera of infected TB patients and their contacts mounted significantly higher antibody responses against Rv1509 signature protein than those of healthy controls (**Figure 7E**) (P<0.0058 for PTB and P<0.0001 for relapse PTB cases). These results point to the likely utility of signature protein Rv1509 as a pre-screening biomarker in tuberculosis.

## Discussion

About 25% of *M.tb* coding genes are still classified as “hypothetical” or “uncharacterized”, making it imperative to dissect the functions of these hypothetical proteins, which could improve our understanding of *M.tb* virulence and pathogenesis. In this study, the ‘Signature protein’ was identified by comparing protein sequences of 13 different mycobacterium species using BLAST tool. Interestingly, we found that Rv1509 signature protein is a unique protein exclusively present in *M.tb* and absent in other mycobacterial species, even BCG. *In silico* analysis revealed that Rv1509 protein is highly antigenic and displays T cell and B cell epitopes. Rv1509 protein was found to have high affinity T cell epitopes. The antigen presentation to T cells *via* MHC class molecules plays an important role in inducing an effective immune response against bacteria (42). Molecular docking results confirmed that the predicted T cell epitopes of Rv1509 interact with HLA*A alleles of MHC Class I and Class II molecules. Further, B-cell response is a crucial part of the adaptive immune system and provides a significant contribution towards protection against *M.tb*. B cells have been shown to modulate the immune response against *M.tb* by shaping T cell-mediated immunity (43). Using immunoinformatic tools, we predicted linear and conformational B cell epitopes in Rv1509. We found a total of 18 B cell epitopes in Rv1509. The presence of high affinity T cell and B cell epitopes suggested its probable role in modulation of adaptive immune responses.

Macrophages and DCs constitute the first line of defense against *M.tb* infection. Macrophages serve as an important niche for the growth and survival of mycobacterial species. Antigen presenting cells (APCs) upon activation initiate a series of cascade of events leading to immunomodulation in terms of inducing innate and acquired host immune response. Interestingly, we found that treatment of macrophages with Rv1509 increases expression of activation markers such as co-stimulatory marker CD80, MHC-I and MHC-II, indicating a role of Rv1509 in inducing innate immune response. Then, we tested the levels of cytokines produced by activated macrophages. We found that macrophages treated with Rv1509 showed an increase in the secretion of inflammatory cytokines like TNF*α*, IL-6 and IL-12 compared to untreated cells. DC’s are potent APCs that can activate naïve T cells to initiate an immune response against pathogens, including *M.tb*. One of the mechanisms by which *M. tb* successfully subverts host immune attack is by interfering with the functions of DCs (44, 45). *M.tb* impairs DC maturation, thereby delaying T cell response. Therefore, we investigated the effect of Rv1509 protein on DC maturation. Interestingly, Rv1509 treated DCs displayed significant up-regulation of activation markers such as CD80 and MHCII, denoting mature phenotype that could effectively stimulate T cells. DC-SIGN, an important *M tb* receptor present on DCs is vital for *M.tb* and DC interactions, leading to successful invasion and infection by *M.tb* inside the host cells (41, 45). Intriguingly, Rv1509 protein downregulated the expression levels of DC-SIGN marker, suggesting Rv1509 to be favoring host defense mechanisms against *M.tb* infection. Besides, DC-SIGN downregulation is also a marker for DC maturation as mature DCs are known to have low phagocytic ability as compared to immature DC’s. These observations strongly argue for a possible role of Rv1509 as a vaccine candidate against TB. Cellular immune responses controlled by CD4+/CD8+T cells form the central element of the adaptive immunity against *M.tb*. CD4+T cells are important during the acute phase of infection, whereas CD8+T cells play a crucial role in the clearance of infection during the chronic phase. The optimal cross-talks between APCs such as DCs and T cells play a significant role in controlling *M.tb* infection. Our results displaying Rv1509 promoting macrophage activation and DC maturation, suggested effective activation of innate immune response that positively modulates the adaptive immune system. Thus, we analyzed the impact of Rv1509 protein on the production and expansion of polyfunctional IFN *γ* and TNF*α* secreting CD4+and CD8+T cells. The polyfunctional CD4+T cells is an important immune correlate of protection against TB (46). The percentage of polyfunctional CD4+ and CD8+T cells in mice immunized with Rv1509 displayed a significant increase as compared to mice injected with the PBS buffer only. These findings indicate the possible role of Rv1509 in inducing host protective immune response, having implications in vaccine development against *M.tb.* However, induction of polyfunctional CD4+T cells does not necessitate protection against *M.tb*, as other attributes such as memory response and differentiation of T cells may be equally important in conferring protection against TB (46). Though FACS plots marginally suggest reduction in TNFα single positive cells but it will be too premature to comment on that as we observed significant increase in secretory TNF-α in culture supernatants after protein treatment (**Figure 4C**). One of the contributing factors towards the reduction in TNF-α single positive cells could be concomitant increase in polyfunctional cells (TNF-α+ IFN-γ+) upon treatment with Rv1509. The supplementary reasons are open to exploration and will be probed further in subsequent follow-up studies.

T cells can mediate their immune response through different subsets, including Th1, Th2, Th17, Treg and T_FH_ cells. Among these subsets, Th1 type of immune response is primarily responsible for protective immunity against *M.tb*. Th1 cells are involved in containment and control of *M.tb* replication and involve the production of pro-inflammatory cytokines, mainly IL-2, IFN*γ* and TNF*α* (47). Splenocytes stimulated with Rv1509 protein showed an enhanced secretion of pro-inflammatory Th1 cytokines including IFNγ, IL1β and IL-6. Therefore, mycobacterial antigens capable of boosting Th1 immune response have the potential to be considered as vaccine candidates, although, Th1 based cytokine production does not confer complete protection against TB in humans (47). Moreover, a substantial effector memory response in splenocytes stimulated with Rv1509 also indicated that Rv1509 might act as a recall antigen in a Th1 memory response to counter recurrent infection. Even though the memory phenotype was evaluated at short term, these results were encouraging considering the inefficacy of BCG to evoke the memory response as one of the reasons of its impotency.

In recent years, B cells have emerged as critical players in mediating protection against tuberculosis. B cell executes different immunological functions, including antibody response, cytokine production, as well as antigen presentation (48). In line with our previous *in silico* findings, we were curious to determine Rv1509-mediated B cell response in terms of measuring IgG reactivity. Sera from immunized mice showed an increase in IgG levels compared to the controls. To get further hints in human subjects, we assessed the sera of TB patients for antibodies (IgG) against Rv1509. We found a non-overlapping significant IgG immunoreactivity in human TB patients compared to healthy controls, pointing to the fact that response to Rv1509 is specific, confirming our observation that this protein is exclusively present in *M.tb*. Despite the major impetus on cell mediated immunity for control against TB, recent observations have revealed critical role of B cells in TB control. B cells effectively modulate and shape the T cell mediated immune response *via* antigen presentation or cytokine release. Intriguingly B cells also help in development of T cell memory and protection against a secondary challenge. These observations highlight a new arena wherein vaccines that target both cell mediated and humoral response are more effective.

The dilemma was to decipher how this signature protein could perform so many functions. There have been a few studies which have attempted to understand the mechanisms by which a protein can perform multiple functions. Rv1509 may undergo different folding/refolding pathway in response to changes in the environment, leading to conformational changes and different functions (49, 50). Another possible way is that Rv1509 undergoes post-translational modifications leading to its moonlighting functions. Also, the intrinsically disordered regions of Rv1509 may play a pivotal role in performing important roles in protein function. It has been previously shown that intrinsically disordered regions in proteins may undergo a conformational change to facilitate different functions of a protein, including immune modulation (51). Besides, the cysteine residues present in Rv1509 can also contribute to modulating protein functions by changing their redox state, which involves changes in intracellular/intercellular disulphide bond (52).

Taken together, we describe the properties of a hypothetical protein, Rv1509, present exclusively in *M.tb* and absent in all other mycobacterial species including BCG. The host targets this protein by exploiting it to mount a strong pro-host immune response including an effector memory response as evident from a series of experiments involving *in vitro* studies, mice studies and sera of TB patients. These findings highlight the host-pathogen tussle for their individual existence, and the outcome is dependent on a likely complex set of interactions with other microbial/host factors. Our findings point towards Rv1509 protein as a probable vaccine candidate as it showed promising immunomodulatory functions. Further studies in *M.tb* would shed more insights into understanding the function(s) of Rv1509 in virulence and pathogenesis.

## Data Availability Statement

The original contributions presented in the study are included in the article/**Supplementary Material**. Further inquiries can be directed to the corresponding authors.

## Ethics Statement

The studies involving human participants were reviewed and approved by Institute ethics committee, AIIMS, New Delhi. The patients/participants provided their written informed consent to participate in this study. The animal study was reviewed and approved by Institute animal ethics committee, National Institute of Pathology, New Delhi.

## Author Contributions

NE, SR, and SH conceived and designed all experiments. MP, JA, JS, JAS, SA, MK, SR, IK and HA performed the experiments. MP, JA, NE, and SH wrote the manuscript with inputs from SR, JAS, and KL. JAS, AS, KL, and GS helped in interpretation of results, and JAS edited the final manuscript. All authors contributed to the article and approved the submitted version.

## Funding

SH and NE thank the Department of Biotechnology (DBT), Ministry of Science and Technology, Government of India (MoS&T, GoI) for DBT North-East Grants (BT/PR23099/NER/95/632/2017) and (BT/PR23155/NER/95/634/2017) by Department of Biotechnology, Ministry of Science and Technology (MoS&T), Government of India (GoI). The work was partly funded by the Indian Council of Medical Research (ICMR) Intramural Grant.

## Conflict of Interest

Author SAR was employed by company BioInceptionPvt. Ltd.

The remaining authors declare that the research was conducted in the absence of any commercial or financial relationships that could be construed as a potential conflict of interest.

## Publisher’s Note

All claims expressed in this article are solely those of the authors and do not necessarily represent those of their affiliated organizations, or those of the publisher, the editors and the reviewers. Any product that may be evaluated in this article, or claim that may be made by its manufacturer, is not guaranteed or endorsed by the publisher.

## Supplementary Material

Supplementary Figure 1 Computational analysis of 25 unique proteins. **(A)** Grand average of hydropathy. **(B)** Instability index of Unique Proteins. Red color signifies stable proteins whereas Blue color signifies unstable proteins. The hypothetical protein Rv1509 is in unstable category. **(C)** Secondary structure prediction showing number of alpha-helix and beta sheets. **(D)** Analysis of globular domains using GlobPlot prediction tool. **(E)** Western blot showing the expression of Rv1509 in different *M.tb* fractions (10µg/lane).Majority of the Rv1509 protein is detected in total membrane fraction and some amount is present in the cytosol fraction of *M.tb.* **(F)** The SDS PAGE image of expression of Rv1509 protein in Clear Coli cells after purification by Column Chromatography.

Supplementary Figure 2 Conformational B cell epitopes. The epitope is shown in ball model and receptor is represented in sticks in cyan blue color. **(A)** Peptide 1, 33-38_LRSLFD. **(B)** Peptide 2, 1-9_VFALSNNLN. **(C)** Peptide 3, 275-284_AGYVPATLQP. **(D)** Peptide 4, 56-61_ARLPVG. **(E)** Peptide 5, 169-172_VFPY. **(F)** Peptide 6, 196-202_EGNTGMD.

Supplementary Figure 3 Purity of monocyte derived dendritic cells. Monocytes were isolated from PBMCs after density gradient centrifugation and seeded in 24 well plates. The cells were treated with GM-CSF and IL4 to mature them to Dendritic Cells. Further cells were collected and stained with CD11c antibody to check the purity of the DCs followed by FACS analysis. **(A)** The plot shows the purity of monocyte derived dendritic cells. **(B)** Monocytes treated with GM-CSF and IL4. The red color in panel A symbolizes population negative for CD11c marker. The green color in panel B symbolizes population positive for CD11c marker. **(C)** RAW 264.7 macrophages were seeded in 96 well (50×10^3^ cells/well) followed by treatment with different concentration of Rv1509 protein (1, 2, 4 and 8μg/ml). MTT was added (10X) to the cells along with media and incubated for 3 hours in dark followed by addition of 200μl DMSO. The absorbance was taken and percentage of cell death was calculated against untreated cells. The bar graphs represent the cytotoxicity of Rv1509 protein at different concentrations in RAW cells using MTT assay after 48 hrs treatment. Data represents samples plated in triplicates from a representative experiment performed at least three times.

Supplementary Figure 4 **(A)** The molecular interactions analysis of TLR4 and Rv1509 shows that the protein interacts with several amino acid residues of the binding pocket of TLR2 as shown in the figure. **(B–D)** Secretion of pro inflammatory cytokines IL-6, IL-12 and TNFα upon stimulation with Rv1509 protein. Macrophages (RAW264.7, TLR4 knockout and TLR2/4 double knockout)were treated with Rv1509 protein at different concentrations (1, 2 and 4μg/ml) for 48 hrs. Supernatants were collected and ELISA was performed for determination of IL-6, IL-12 and TNFα levels using BD ELISA kits. No cytokine release is observed from the cells that were genetic mutants for TLR2.

Supplementary Figure 5 Gating strategy for FACS analysis. **(A)** From the whole cell population lymphocytes were separated based on their size and where these cells were embedded. **(B)** From the Lymphocyte population CD3 cells were selected based on the fluorophore against side scattered area. **(C)** CD4+ and CD8+ cell populations were separated through gating from the total CD3 population and further gating was done for the CD44 and CD62L from individual CD4+ and CD8+ cells. **Figures S5A–C** represent gating strategy for CD44 and CD62L. **(D)** For polyfunctional T cell gating we selected whole lymphocyte population from the total cells used for FACS analysis. **(E)** This figure represents the gating of CD4+ and CD8+ cells. From total lymphocyte population CD4+ and CD8+ cells were gated on their respective panels.
