# Supplementary figures and images for: Mycobacterium tuberculosis Specific Protein Rv1509 Evokes Efficient Innate and Adaptive Immune Response Indicative of Protective Th1 Immune Signature
Source: Front Immunol. 2021 Jul 27;12:706081. doi: 10.3389/fimmu.2021.706081 (PMC8354026; doi:10.3389/fimmu.2021.706081)

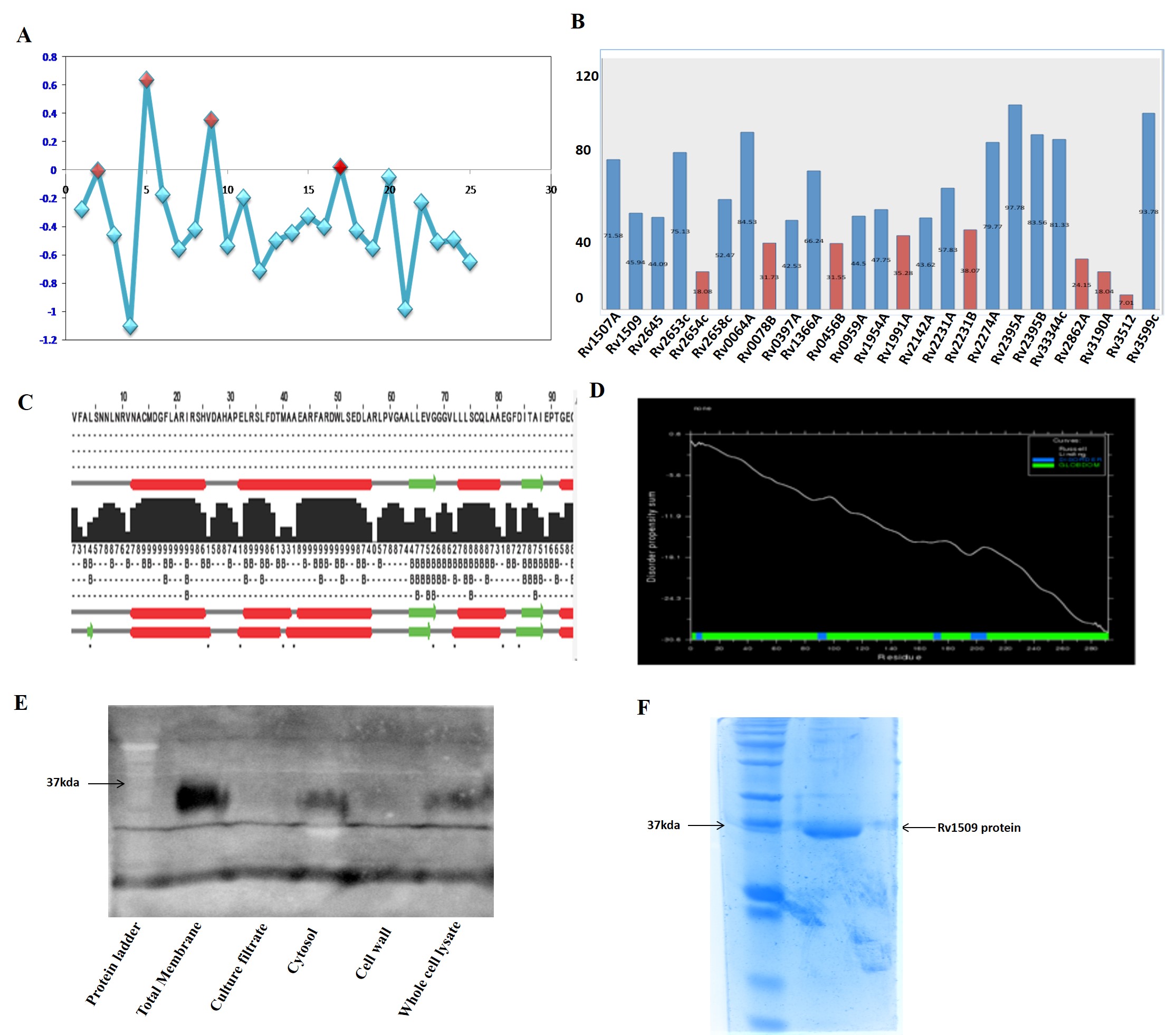

Supplement: Supplementary Figure 1 — Computational analysis of 25 unique proteins. (A) Grand average of hydropathy. (B) Instability index of Unique Proteins. Red color signifies stable proteins whereas Blue color signifies unstable proteins. The hypothetical protein Rv1509 is in unstable category. (C) Secondary structure prediction showing number of alpha-helix and beta sheets. (D) Analysis of globular domains using GlobPlot prediction tool. (E) Western blot showing the expression of Rv1509 in different M.tb fractions (10µg/lane).Majority of the Rv1509 protein is detected in total membrane fraction and some amount is present in the cytosol fraction of M.tb. (F) The SDS PAGE image of expression of Rv1509 protein in Clear Coli cells after purification by Column Chromatography. [file Image_1.jpeg]

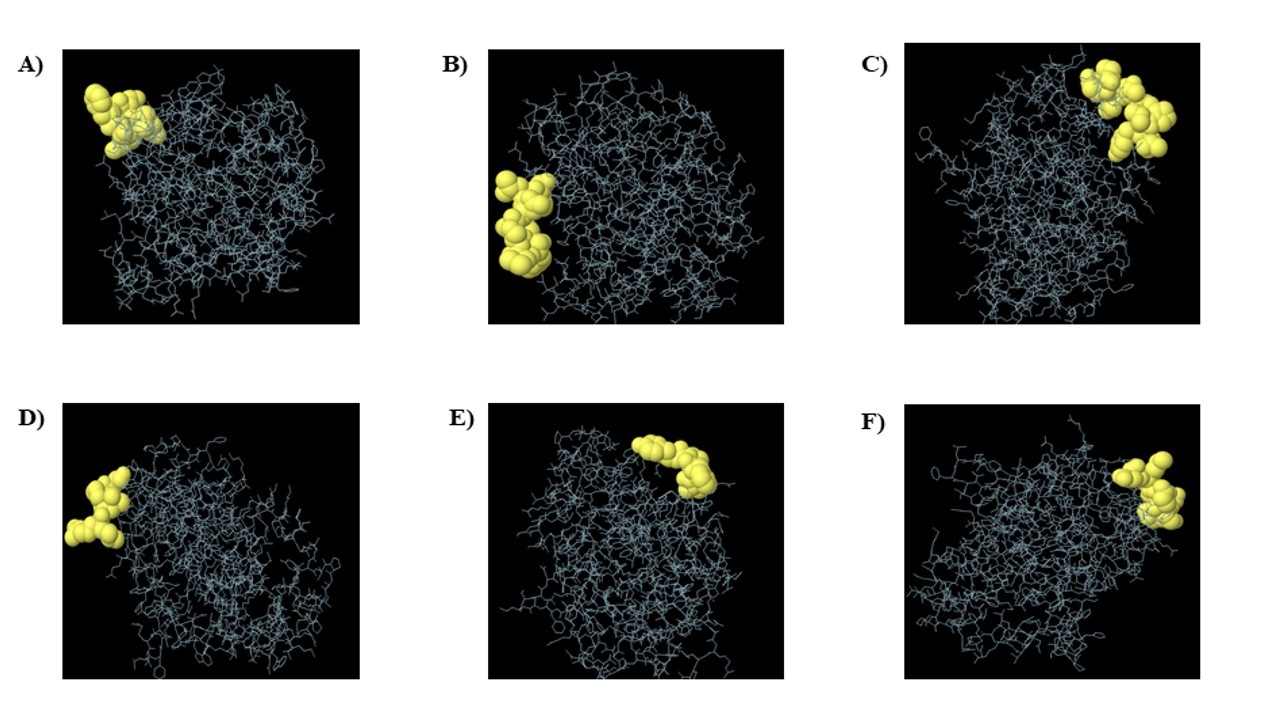

Supplement: Supplementary Figure 2 — Conformational B cell epitopes. The epitope is shown in ball model and receptor is represented in sticks in cyan blue color. (A) Peptide 1, 33-38_LRSLFD. (B) Peptide 2, 1-9_VFALSNNLN. (C) Peptide 3, 275-284_AGYVPATLQP. (D) Peptide 4, 56-61_ARLPVG. (E) Peptide 5, 169-172_VFPY. (F) Peptide 6, 196-202_EGNTGMD. [file Image_2.jpeg]

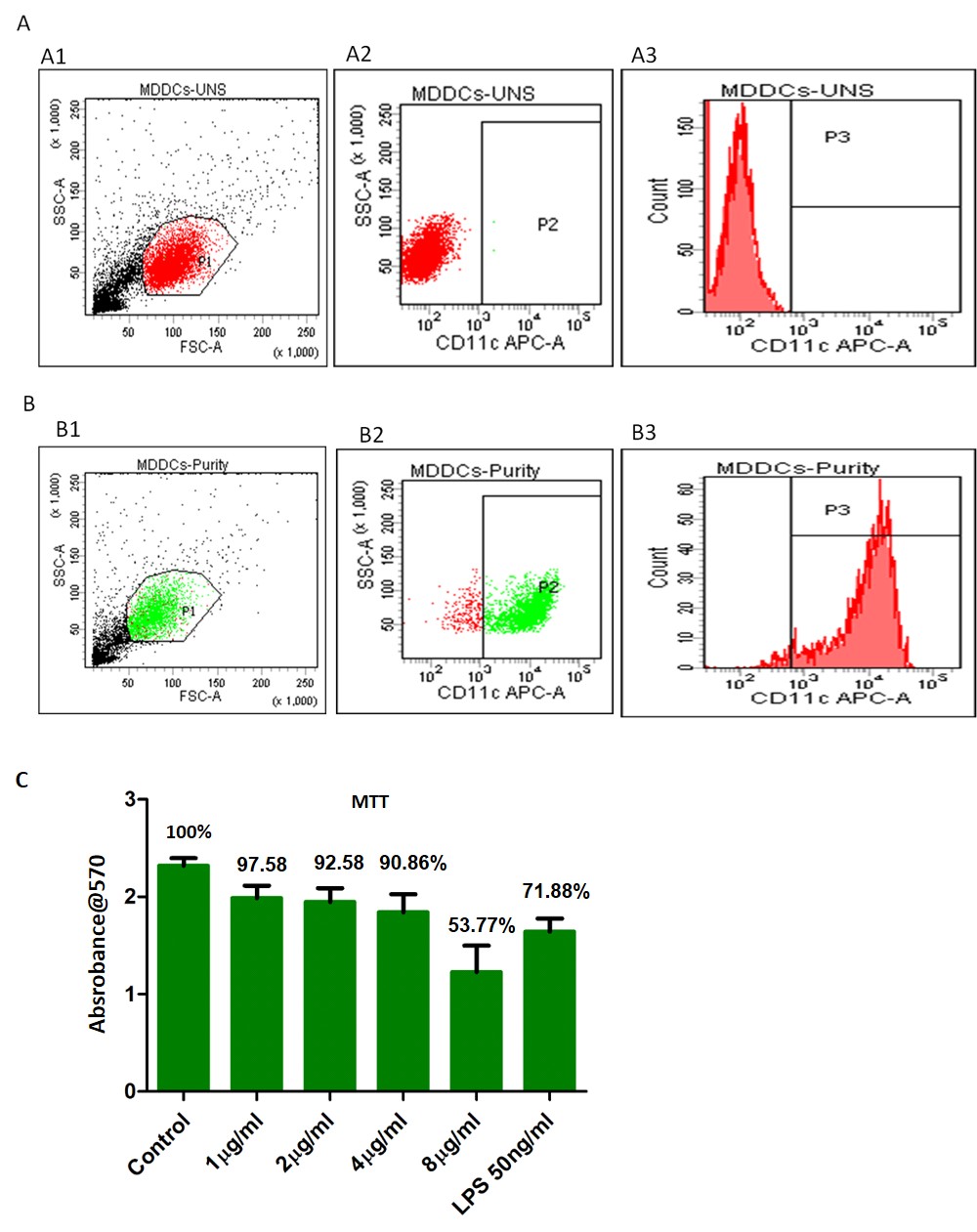

Supplement: Supplementary Figure 3 — Purity of monocyte derived dendritic cells. Monocytes were isolated from PBMCs after density gradient centrifugation and seeded in 24 well plates. The cells were treated with GM-CSF and IL4 to mature them to Dendritic Cells. Further cells were collected and stained with CD11c antibody to check the purity of the DCs followed by FACS analysis. (A) The plot shows the purity of monocyte derived dendritic cells. (B) Monocytes treated with GM-CSF and IL4. The red color in panel A symbolizes population negative for CD11c marker. The green color in panel B symbolizes population positive for CD11c marker. (C) RAW 264.7 macrophages were seeded in 96 well (50×103 cells/well) followed by treatment with different concentration of Rv1509 protein (1, 2, 4 and 8μg/ml). MTT was added (10X) to the cells along with media and incubated for 3 hours in dark followed by addition of 200μl DMSO. The absorbance was taken and percentage of cell death was calculated against untreated cells. The bar graphs represent the cytotoxicity of Rv1509 protein at different concentrations in RAW cells using MTT assay after 48 hrs treatment. Data represents samples plated in triplicates from a representative experiment performed at least three times. [file Image_3.jpeg]

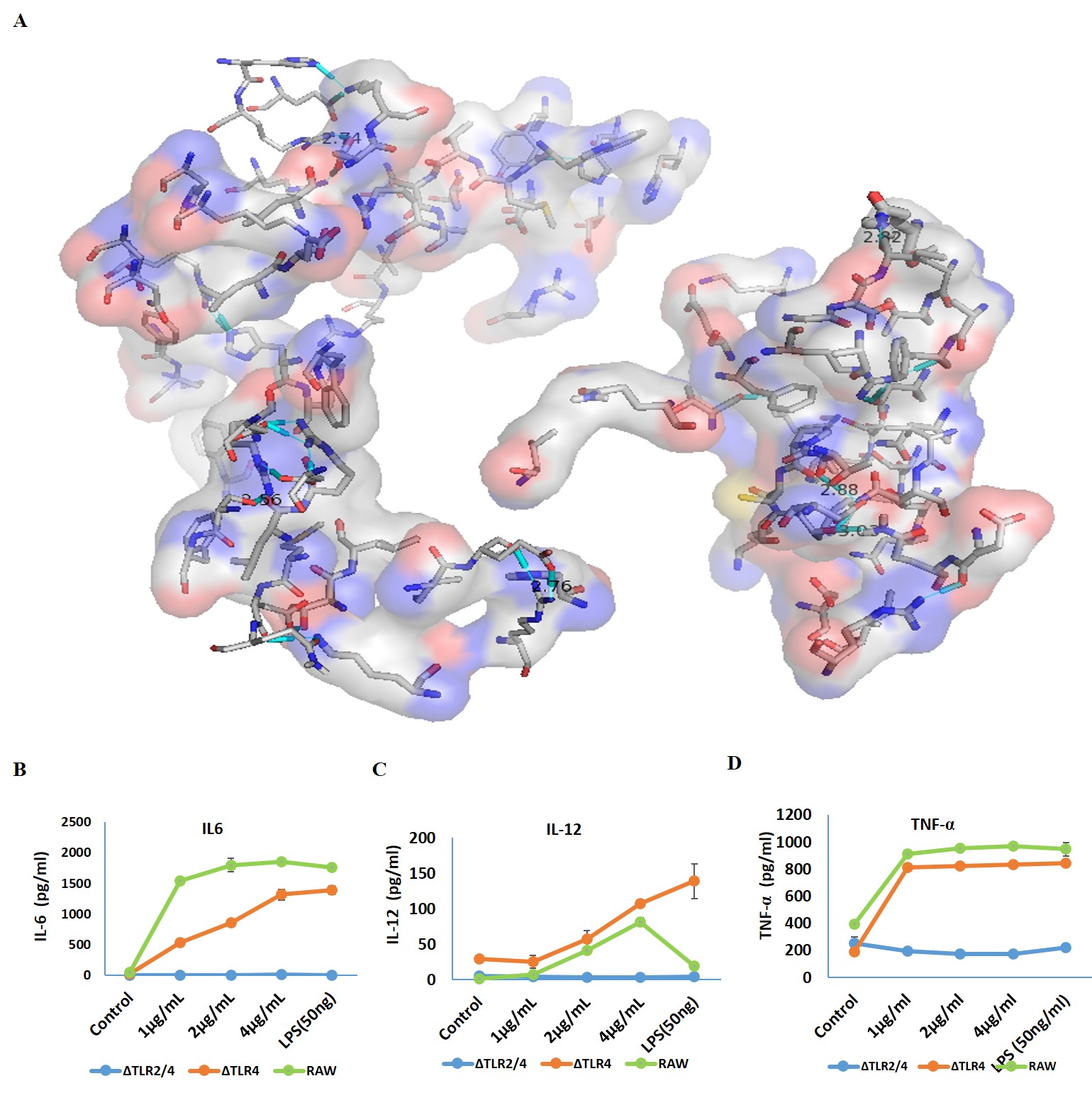

Supplement: Supplementary Figure 4 — (A) The molecular interactions analysis of TLR4 and Rv1509 shows that the protein interacts with several amino acid residues of the binding pocket of TLR2 as shown in the figure. (B–D) Secretion of pro inflammatory cytokines IL-6, IL-12 and TNFα upon stimulation with Rv1509 protein. Macrophages (RAW264.7, TLR4 knockout and TLR2/4 double knockout)were treated with Rv1509 protein at different concentrations (1, 2 and 4μg/ml) for 48 hrs. Supernatants were collected and ELISA was performed for determination of IL-6, IL-12 and TNFα levels using BD ELISA kits. No cytokine release is observed from the cells that were genetic mutants for TLR2. [file Image_4.jpeg]

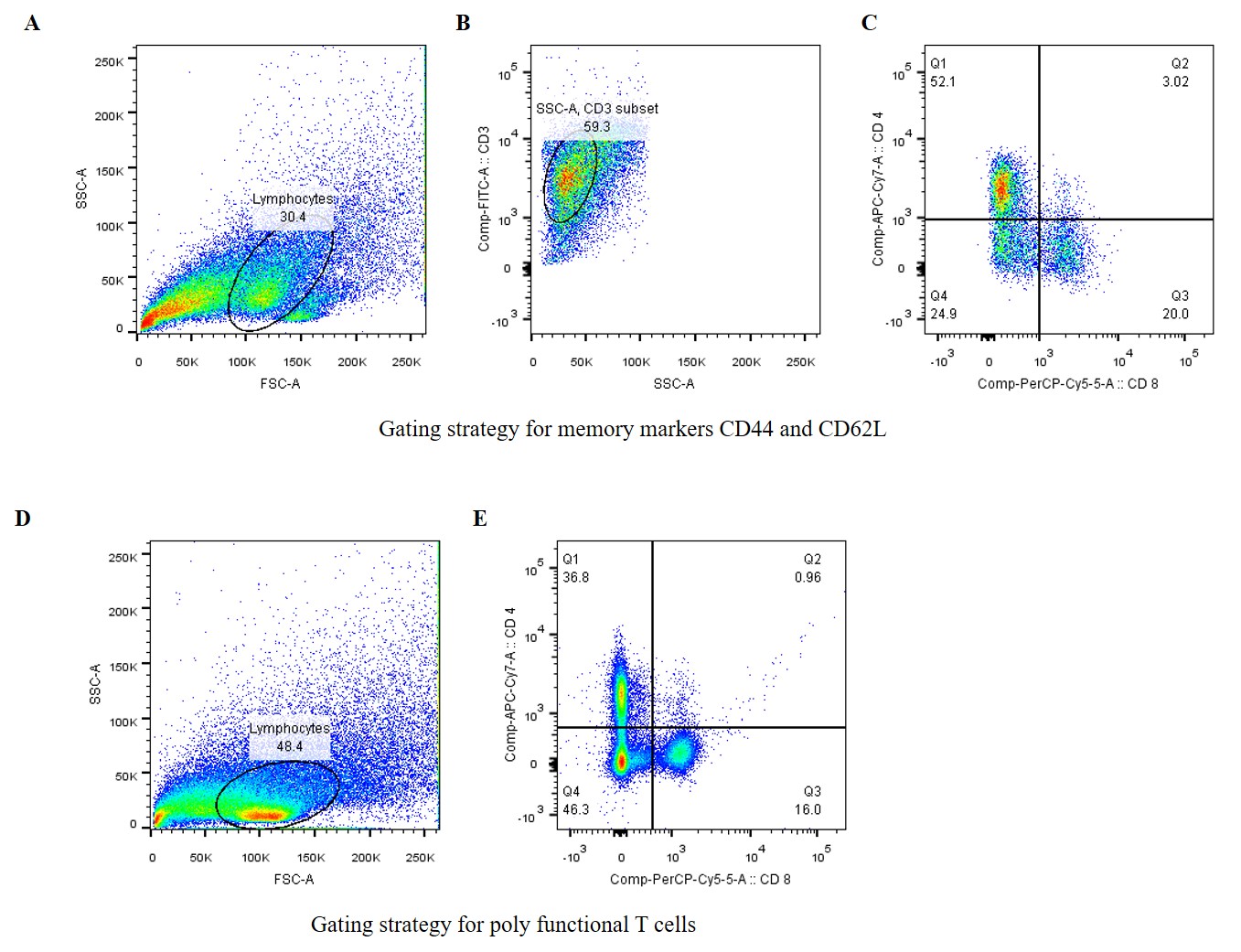

Supplement: Supplementary Figure 5 — Gating strategy for FACS analysis. (A) From the whole cell population lymphocytes were separated based on their size and where these cells were embedded. (B) From the Lymphocyte population CD3 cells were selected based on the fluorophore against side scattered area. (C) CD4+ and CD8+ cell populations were separated through gating from the total CD3 population and further gating was done for the CD44 and CD62L from individual CD4+ and CD8+ cells. Figures S5A–C represent gating strategy for CD44 and CD62L. (D) For polyfunctional T cell gating we selected whole lymphocyte population from the total cells used for FACS analysis. (E) This figure represents the gating of CD4+ and CD8+ cells. From total lymphocyte population CD4+ and CD8+ cells were gated on their respective panels. [file Image_5.jpeg]
