# Supplementary material for: Mycobacterium tuberculosis Specific Protein Rv1509 Evokes Efficient Innate and Adaptive Immune Response Indicative of Protective Th1 Immune Signature
Source: Front Immunol. 2021 Jul 27;12:706081. doi: 10.3389/fimmu.2021.706081 (PMC8354026; doi:10.3389/fimmu.2021.706081)
Supplement: Supplementary file 6 [file DataSheet_1.docx]

**Supplementary Table 1**: List of antibodies used in this study.

**
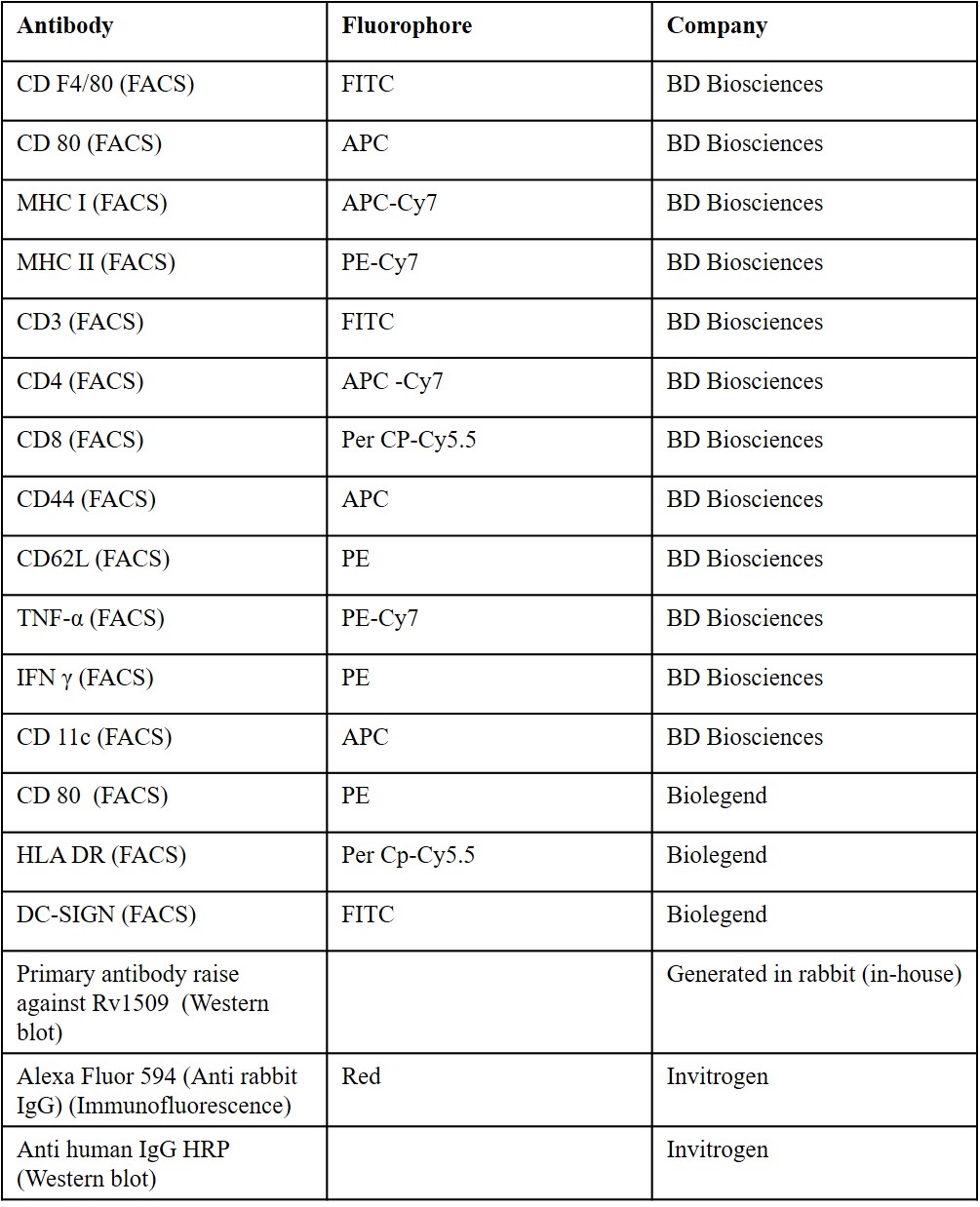
**

**Supplementary Table 2**: Different species of Mycobacteria selected for computational analysis.


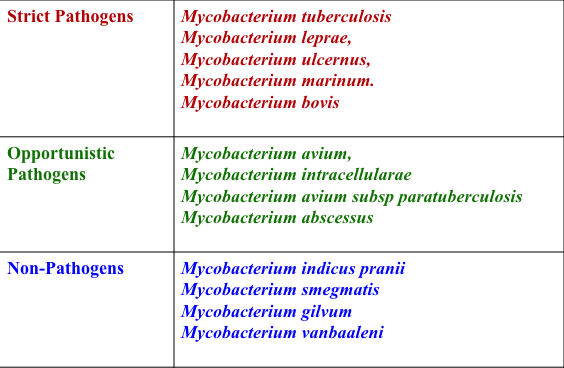


**Supplementary Table 3:** Unique proteins identified from comparative analysis of all protein sequences present in 13 species of Mycobacteria.

| **H_37_Rv Gene Identifiers** | **H_37_Rv protein description** |
| --- | --- |
| **Rv1507A** | Hypothetical protein |
| **Rv1509** | Hypothetical protein |
| **Rv2645** | Hypothetical protein |
| **Rv2653c** | Possible PhiRv2 prophage protein |
| **Rv2654c** | Possible PhiRv2 prophage protein |
| **Rv2658c** | Possible prophage protein |
| **Rv0064A** | Possible antitoxin VapB1 |
| **Rv0078B** | Hypothetical protein |
| **Rv0397A** | Hypothetical protein |
| **Rv0456B** | Possible antitoxin MazE1 |
| **Rv0959A** | Possible antitoxin VapB9 |
| **Rv1366A** | Hypothetical protein |
| **Rv1954A** | Hypothetical protein |
| **Rv1991A** | Antitoxin MazE6 |
| **Rv2142A** | Possible antitoxin ParD2 |
| **Rv2231A** | Possible toxin VapC16 |
| **Rv2231B** | Possible antitoxin VapB16 |
| **Rv2274A** | Possible antitoxin MazE8 |
| **Rv2395A** | Acid and phagosome regulated protein AAprA |
| **Rv2395B** | Acid and phagosome regulated protein B AprB |
| **Rv2862A** | Possible antitoxin VapB23 |
| **Rv3190A** | Hypothetical protein |
| **Rv3344c** | PE-PGRS family protein PE_PGRS49] [partial=5'] |
| **Rv3512** | PE-PGRS family protein PE_PGRS56] [partial=5'] |
| **Rv3599c** | Hypothetical short protein |

**Supplementary Table 4:** A list of proteins which are unique to *M. tb* at both protein and nucleotide level. Nucleotides sequences of proteins which show more than 90% similarity in BCG are marked as green while those marked in red are unique to *M.tb.*


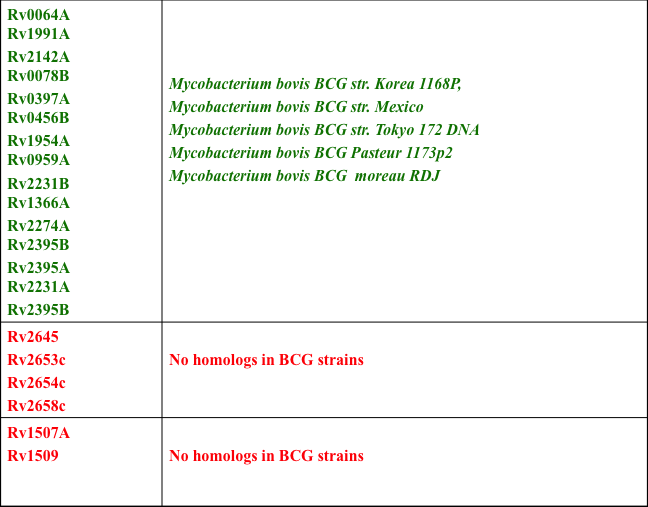


**Supplementary Table 5:** Predicted MHC-II T cell binding epitopes of Rv1509

| S.No | Start | End | Peptide | Length | smm_align_IC50 |
| --- | --- | --- | --- | --- | --- |
| 1 | 253 | 267 | QWMV**AAIRSAVKL**RV | 15 | 199 |
| 2 | 258 | 272 | WMVAAIRSAVKLRVH | 15 | 141 |
| 3 | 260 | 274 | VAAIRSAVKLRVHHL | 15 | 96 |
| 4 | 261 | 275 | AAIRSAVKLRVHHLA | 15 | 97 |
| 5 | 255 | 269 | RAQWMVAAIRSAVKL | 15 | 195 |
| 6 | 254 | 268 | RRAQWMVAAIRSAVK | 15 | 198 |

**Supplementary Table 6:** Predicted MHC-I T cell binding epitopes of Rv1509

| S.No | Start | End | Peptide | Length | IC50 |
| --- | --- | --- | --- | --- | --- |
| 1 | 277 | 285 | **YVPATLQPI** | 9 | 4.85 |
| 2 | 259 | 267 | MVAAIRSAV | 9 | 16.6 |
| 3 | 168 | 176 | Y**VFPY**EPHF | 9 | 18.69 |
| 4 | 261 | 269 | **AAIRSAVKL** | 9 | 26.73 |

**Supplementary Table 7:** Predicted linear B cell epitopes of Rv1509 protein

| S.No | Start | End | Peptide | Length |
| --- | --- | --- | --- | --- |
| 1 | 5 | 13 | SNNLNRVNA | 9 |
| 2 | 24 | 38 | SHVDAHAPELRSLFD | 15 |
| 3 | 52 | 58 | SEDLARL | 7 |
| 5 | 99 | 113 | RQLGDIVLELAAARP | 15 |
| 6 | 118 | 127 | CKAEDFISEK | 10 |
| 8 | 170 | 180 | FPYEPHFNIPT | 11 |
| 9 | 191 | 218 | MRHRIEGNTGMDDPKGVWRSLNWITVPK | 28 |
| 10 | 224 | 234 | AKDATLTLRFH | 11 |
| 11 | 245 | 257 | ALTDKEFAGRRAQ | 13 |
| 12 | 268 | 272 | KLRVH | 5 |
| 13 | 283 | 284 | QP | 2 |

**Supplementary Table 8:** Predicted conformational B cell epitopes of Rv1509

| S.No | Start | End | Peptide | Length | Score |
| --- | --- | --- | --- | --- | --- |
| 1 | 33 | 38 | LRSLFD | 6 | 0.878 |
| 2 | 1 | 9 | VFALSNNLN | 9 | 0.86 |
| 3 | 275 | 284 | AG**YVPATLQP** | 10 | 0.858 |
| 4 | 56 | 61 | ARLPVG | 6 | 0.834 |
| 5 | 169 | 172 | **VFPY** | 4 | 0.814 |
| 6 | 196 | 202 | EGNTGMD | 7 | 0.81 |
